# Clinical Significance of Carotid Intima-Media Complex and Carotid Plaque Assessment by Ultrasound for the Prediction of Adverse Cardiovascular Events in Primary and Secondary Care Patients

**DOI:** 10.3390/jcm10204628

**Published:** 2021-10-09

**Authors:** Anna Kabłak-Ziembicka, Tadeusz Przewłocki

**Affiliations:** 1 Department of Interventional Cardiology, Institute of Cardiology, Jagiellonian University Medical College, 31-202 Krakow, Poland; 2 Noninvasive Cardiovascular Laboratory, John Paul II Hospital, Prądnicka 80, 31-202 Krakow, Poland; 3 Department of Cardiac and Vascular Diseases, Institute of Cardiology, Jagiellonian University Medical College, John Paul II Hospital, 31-202 Krakow, Poland; tadeuszprzewlocki@op.pl; 4 Department of Interventional Cardiology, John Paul II Hospital, Prądnicka 80, 31-202 Krakow, Poland

**Keywords:** cardiovascular risk, carotid intima-media complex, carotid plaque, major adverse cardiac and cerebral events, prevention, scores

## Abstract

Recently published recommendations from the American Society of Echocardiography on ‘Carotid Arterial Plaque Assessment by Ultrasound for the Characterization of Atherosclerosis and Evaluation of Cardiovascular Risk’ provoked discussion once more on the potential clinical applications of carotid intima-media complex thickness (CIMT) and carotid plaque assessment in the context of cardiovascular risk in both primary and secondary care patients. This review paper addresses key issues and milestones regarding indications, assessment, technical aspects, recommendations, and interpretations of CIMT and carotid plaque findings. We discuss lacks of evidence, limitations, and possible future directions.

## 1. Introduction

Cardiovascular disease (CVD) is a leading global problem [1]. An estimated 17.9 million people died from CVDs in 2019, representing 32% of all global deaths [1]. Of these deaths, 85% were due to major adverse cardiac and cerebral events (MACCE) [1]. Atherosclerosis and its complications, i.e., MACCE, heart failure, disability, vascular dementia, renal failure, lower limb ischemia, etc. are responsible for more than 50% of all deaths in westernized societies [2]. According to the WHO targets, it is important to detect CVD as early as possible so that management with counseling and medicine can begin [1].

As atherosclerosis is a generalized disease affecting many arterial beds at the same time, assessment of carotid arteries theoretically creates a unique opportunity to mirror and track atherosclerotic disease [2,3,4,5,6,7].

Recently published recommendations from the American Society of Echocardiography on ‘Carotid Arterial Plaque Assessment by Ultrasound for the Characterization of Atherosclerosis and Evaluation of Cardiovascular Risk’ [8], once more opened discussion on the potential clinical applications of carotid intima-media complex thickness (CIMT) and carotid plaque assessments in the context of cardiovascular (CV) risk in both primary and secondary care patients.

CIMT assessment, with or without carotid plaque inclusion, was considered a surrogate measure of atherosclerosis to provide information on the CV outcome in asymptomatic patients with CV risk factors and patients with known atherosclerotic disease, or to measure the effect of medical therapy [8,9,10,11,12,13,14,15,16,17,18,19,20,21,22,23,24,25,26,27,28,29,30,31,32,33,34,35,36,37,38,39,40,41,42].

In epidemiological studies in asymptomatic individuals, increased CIMT values indicate higher risk of stroke, myocardial infarction (MI), or CV mortality [10]. The identification of carotid plaque even enhances this risk [8,11]. Carotid plaque presence, its number and size, volume, surface, echogenicity, or vascularization are all possible measures of MACCE [12,13,14,15,16,17,18,19,20]. Additionally, in secondary prevention for high-risk patients who already suffered from MI or stroke, the carotid atherosclerosis burden may have a role in the prediction of recurrent MACCE [21,22,23,24,25].

Despite so many advantages, the main flaw of CIMT and carotid plaque assessments is the heterogeneous techniques used for taking measurements, data reproducibility, and the limitations on the plaque composition judgements.

Therefore, this review paper addresses key issues and milestones regarding the indications, assessment, technical aspects, and interpretation of CIMT and carotid plaque findings. We discuss lacks of evidence, limitations, and possible future directions.

## 2. Historical Rationale for CIMT Assessment

The conception of CIMT and carotid plaque assessment dates back to the late years of the 20th century [43,44]. Its introduction was closely related to the development of high-resolution ultrasound techniques that allowed for the imaging of a double-layer carotid wall structure (intima-media and adventitia) [43,44].

The first report showed a difference in CIMT values between patients with hyperlipidemia as compared to healthy age-matched individuals [44]. In result, ultrasound images of the carotid artery at the level of the common carotid (CIMT-CCA), the carotid bifurcation (CIMT-CB), or the proximal segment of internal carotid artery (CIMT-ICA) were quickly adopted as a surrogate measure for atherosclerosis by epidemiological studies, i.e., the Kuopio Heart Study, the Atherosclerosis Risk in Communities Study, and the Cardiovascular Health Study [45,46,47].

Later, CIMT was recognized as the equivalent of “subclinical” CVD in asymptomatic patients before an individual develops symptoms such as angina, ischemic stroke (IS), or limb ischemia and was validated to predict CV outcomes [12,13,14,15,16,17,18,19,20].

## 3. Normal vs. Abnormal CIMT Value: Carotid Plaque Definitions

CIMT values are age and sex specific, and males have higher CIMT on average compared to females [48,49,50,51,52]. Therefore, normative absolute CIMT values are obsolete, and previous normative CIMT cut-off values such as the 0.9 mm mentioned in the European Society of Cardiology guideline should not be used [53]. Several studies have investigated normal CIMT value ranges, and they are summarized in Table 1.

A CIMT value over the 75th percentile, according to American Society of Echocardiography (ASE), should be considered abnormal [54]. In 2002, the National Cholesterol Education Program (NCEP) Adult Treatment Panel III stated that an elevated CIMT (above 75th percentile for age and sex) could elevate a person with multiple risk factors at higher risk category [55].

In a research study enrolling 24 medical centers, the 75th percentile of the CIMT-CCA distribution was established at 0.5 and 0.51 mm in female and male below 25 years of age, while it was established at 0.80 and 0.83 mm in healthy individuals over 80 years of age, respectively (Table 1) [51]. Later, Randrianarisoa et al. performed an update on normal values for CIMT, including traditional as well as novel cardiovascular risk factors of atherosclerosis progression, like the body fat distribution, metabolic syndrome, subclinical inflammation, insulin resistance, and disturbances in glucose metabolism [52].

## 4. Techniques for CIMT and Carotid Plaque Assessment: Strengths and Weaknesses

B-mode high-resolution ultrasound is a noninvasive technique that provides one of the best methods for the detection of early stages of atherosclerotic disease [10]. Many studies have successfully applied CIMT as a technique to monitor arterial wall alterations based upon its association with CV risk factors, the incident CVD, and the outcome [9,10,11,12,13,14,15,16,17,18,19,20]. CIMT and carotid plaque measurements including mean-maximal or mean-mean CIMT, plaque thickness, area and volume, and plaque score were all used as imaging outcomes [9,10,11,12,13,14,15,16,17,18,19,20,56,57,58,59].

Unfortunately, diverse approaches for measuring CIMT and plaque as well as different cut-offs and acquisition techniques have caused confusion for the interpretation of CIMT and plaque findings [9,10,11,12,13,14,15,16,17,18,19,20]. Thus, technique, the number of segments of the carotid artery tree, the near or far wall, and the use of contrast-enhanced agents are all points for discussion [56].

Another, but not less important issue, is whether measurements can be manual, (semi)-automated, or if computer-assisted analysis software should be used to automatically track the intima-media layer [10,54,59]. Semi-automated edge detection is more often applied in the setting where only the CCA is examined, while manual edge detection is usually applied in the setting where the CB and the ICA are also measured [58].

For example, in the Carotid Atherosclerosis Progression Study (CAPS), CIMT was expressed as the mean CCA at the far wall and in the Cardiovascular Health Study (CHS) as the mean of the maximum at the near and far CCA and ICA, while in the Malmö Diet and Cancer Study (MDCS) and the Kuopio Ischemic Heart Disease Study (KIHD), it was expressed as the maximum CIMT-CCA at the far wall [12,13,14,15,16]. The Rotterdam study presented results as the maximum value from the near and far CCA [17], while the Atherosclerosis Risk in Communities Study (ARIC) measured CIMT as the mean of means at the right and left CCA, CB, and ICA [18,19].

The lack of methodological standardization of CIMT resulted in contraindication (Class III) for CIMT assessment as CV risk modifier from the 2021 ESC Guidelines on CVD prevention in clinical practice [60].

**Figure 1:**
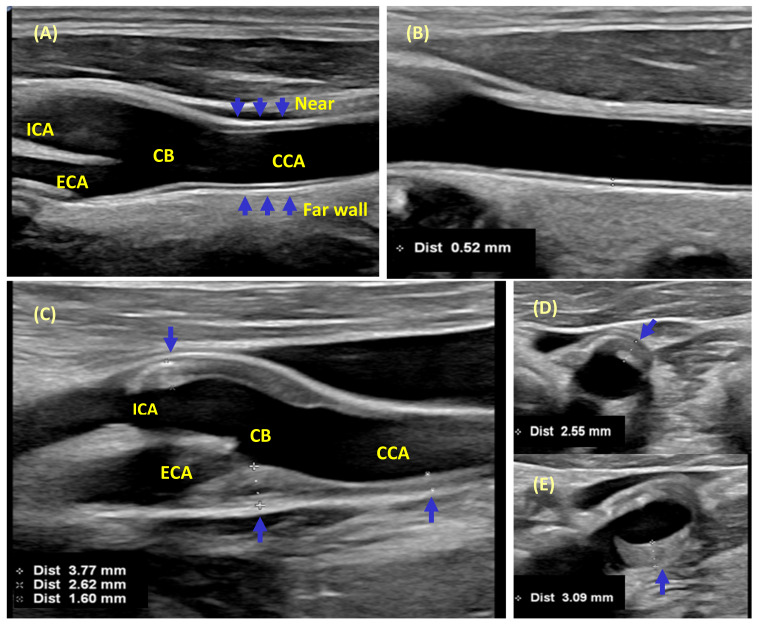
CIMT and carotid plaque assessment. (**A**) CIMT is a double-line pattern on both walls of the common carotid artery (CCA), the carotid bulb (CB), the internal carotid artery (ICA), and the external carotid artery (ECA) in a B-Mode 2D longitudinal image. Two parallel lines are the lumen-intima and media-adventitia interfaces. (**B**) According to the Mannheim IMT consensus [59], CIMT should be measured within a region free of plaque with a clearly identified double-line pattern, preferably on the far wall of the CCA at least 5 mm below its end. If the CIMT-CB and the CIMT-ICA are measured, the results should be reported separately from CIMT-CCA. According to the Mannheim [59], limiting CIMT measurements to the far wall of the CCA and distal 1 cm of each CCA is the preferred strategy. (**C**) Diffuse-type plaques (Grades II or III, and CIMT ≥ 1.5 mm). (**C–E**) In line with the ASE consensus, the maximal plaque height should be measured from the side in which a plaque is detected (unilateral) or from the right and left carotid arterial segments (bilateral), using a caliper placed at the adventitial plane and extending into the center of the lumen to the vessel wall. For the purposes of standardization, this measurement should be taken from the long (**C**) and short axis (**D**,**E**) of the carotid artery. Arrows indicate regions of taking CIMT, plaque measurements.

Thus, to overcome methodological flaws, guidelines for obtaining CIMT and carotid plaque measurements were published with the intention to reduce measurement variability, a key parameter for a high-quality study, statistical power, and sample size determination (Table 2, Figure 1).

The 2008 ASE consensus statement for CIMT is based on the concept of identifying asymptomatic patients at high risk who might be candidates for more intensive, evidence-based medical interventions that reduce CVD risk (Table 2) [54]. The 2008 ASE guidelines recommend measuring CIMT and identifying carotid plaque by ultrasound for refining CVD risk assessment in patients at intermediate CVD risk (FRS 6%–20%) without established coronary heart disease (CHD), peripheral arterial disease (PAD), cerebrovascular disease, diabetes, or abdominal aortic aneurysm. Patients with the following clinical circumstances also might be considered for CIMT and carotid plaque measurement: (1) family history of premature CVD in a first degree relative (men < 55 years old, women < 65 years old); (2) individuals younger than 60 years old with severe abnormalities in a single risk factor (e.g., genetic dyslipidemia) who otherwise would not be candidates for pharmacotherapy; or (3) women younger than 60 years old with at least two CV risk factors.

The Mannheim IMT Consensus [59] recommended CIMT assessment for the initial detection of CHD risk in (1) asymptomatic patients at intermediate risk, (2) in the setting of two or more NCEP risk factors, (3) with metabolic syndrome, (4) with a family history of premature CHD, or (5) with a known coronary artery calcium (CAC) score of zero and FRS 11%–20%. According to Mannheim IMT consensus, measurements may include the CCA, the ICA, or the CB segments. Whereas nearly all patients have their CIMT-CCA imaged, successful imaging of the CIMT-ICA and of the CIMT-CB depends both upon the anatomical topography of the patient and on sonographer’s expertise. Thus, the Mannheim IMT consensus advises rather for CIMT-CCA at far wall measurements than from the whole carotid artery tree. The overview of the Mannheim recommendations is presented in Table 2. The Mannheim definition of plaque was adopted by the 2021 ESC Guidelines on CVD prevention in clinical practice as possible CV risk modifier (Class II-b) [60].

The 2020 ASE recommendations for carotid plaque ultrasound suggested a stepwise approach to CV risk stratification using plaque grading via a focused carotid vascular ultrasound and subsequent 2D or 3D plaque quantification in the assessment of asymptomatic patients at risk (Table 2). In patients presented with symptoms suggestive of CHD but normal non-invasive tests (e.g., stress electrocardiogram, stress echocardiography, stress MRI, and nuclear imaging), patients with atherosclerotic plaques in the carotid artery may benefit from more aggressive medical treatment. In contrast, patients without plaque have an excellent CV prognosis [8]. The 2020 ASE consensus did not focus on CIMT, referring to 2008 ASE recommendations for CIMT assessment [8,54]. 

## 5. Simultaneity of Atherosclerotic Burden across Arterial Beds

Multi-site steno-occlusive arterial disease is invariably associated with worse clinical outcomes [4], accounting for 51% MACCE incidence rate in patients with arterial disease (at least 50% lumen reduction) in either coronary, carotid, and renal and lower extremity arterial territories as compared to 27%, 18%, and 9% in patients with 3-site, 2-site, and 1-site arterial disease at 4 years follow-up, respectively [4]. Furthermore, studies are ongoing about which patients with multi-site atherosclerotic occlusive disease would have decreased risk of MACCE following revascularization, and who would benefit more from medical treatment [61]. 

In the general population of individuals aged 30–79 years, the global prevalence of increased CIMT and carotid plaque is estimated to be 27.6% (16.9% to 41.3%) and 21.1% (13.2% to 31.5%), respectively [62,63]. Multisite arterial disease is common in patients with atherosclerotic involvement in one vascular bed, ranging from 10% to 15% in patients with CAD to 60% to 70% in patients with severe carotid stenosis or PAD [64,65].

At large, screening for asymptomatic disease in additional vascular sites has not been proved to improve prognosis. Nevertheless, the mean-max CIMT values from the CCA, the CB, and the ICA with a cut-off value of 1.30 mm nicely distinguish patients with no steno-occlusive arterial disease or stenosis limited to one arterial territory from individuals with larger arterial territory involvements (odds ratio, OR, 35.9, 95% Confidence Interval, 95% CI, 20 to 65) with a sensitivity of 81.6%, specificity of 88.8%, and positive and negative predictive value of 85.1% and 86.3%, respectively [66]. 

A variety of studies evaluated the relationship between CIMT and presence of atherosclerotic abnormalities in the other territories of the arterial system [67,68,69,70,71,72,73,74,75,76,77]. Most studies demonstrated associations between increasing CIMT value and presence and severity of a significant arterial disease (defined as at least 50% or more lumen reduction): CAD, renal artery stenosis, lower and upper extremity athero-oclussive disease, or the abdominal aorta [67,68,69,70,71,72,73,74,75,76,77]. However, correlations between CIMT with incidence and severity of lesions in the other arterial sites are modest, especially when only CIMT-CCA is reported [78,79,80]. Rohani et al. demonstrated a correlation between the extent of CAD and the mean CIMT-CCA of r = 0.44; Adams et al. demonstrated a correlation between 0.23 and 0.29, while Azarkish et al. demonstrated a correlation between 0.36 and 0.47 [78,79,80]. Interestingly, CIMT can rule out significant CAD in women and patients with degenerative aortic stenosis, e.g., a mean-maximum CIMT value of greater than 1.2 mm was predictive (sensitivity, 73.5%; specificity, 72.7%) of concomitant CAD in patients with aortic stenosis [70,73].

A recent meta-analysis of 89 studies showed moderate correlation between CIMT and severity of CAD (r = 0.60, *p* < 0.001) and the number of diseased vessels (r = 0.49, *p* < 0.001) [67]. Additionally, carotid plaque presence and calcification were less, and lipid-rich necrotic core was highly prevalent in nonsignificant versus significant CAD (*p* < 0.001, *p* = 0.03, *p* < 0.001, respectively) [67]. In another large meta-analysis, including 22 studies, the diagnostic sensitivity and specificity of CIMT for CAD were 0.68 and 0.70, respectively [81].

## 6. CIMT and Carotid Plaque in the Context of Cardiovascular Risk Factors

Various risk factors influence CIMT and carotid plaque, including age, gender, diabetes, dyslipidemia, hypertension, cigarette smoking, genetics, and inflammation [82,83,84]. Song et al. performed a systematic review and meta-analysis of the 75 articles on CIMT, carotid plaque, and carotid stenosis [62]. The influence of CV risk factors for increased CIMT and carotid plaque were 2.71 and 1.79 for age per 10-year increase, 0.49 and 0.55 for female sex, 1.76 and 1.70 for current smoking, 2.23 and 1.45 for diabetes, and 1.55 and 1.75 for hypertension, respectively (Table 3) [62]. Another meta-analysis by Ji et al. of 76 cross-sectional studies that evaluated 11 risk factors showed a pooled OR and 95% CI for the probability of the carotid plaque incidence (the Mannheim definition) to be associated with hypertension, diabetes, dyslipidemia, current smoking, hypertriglyceridemia, LDL-C, hypertriglyceridemia, hyperuricemia, hyperhomocysteinemia, and metabolic syndrome (Table 3) [85]. 

In the NOMAS study that assessed 2D carotid plaque area in 1730 primary care individuals above 39 years old, the associations between carotid plaque and age, smoking, systolic blood pressure, diabetes, LDL-C:HDL-C ratio, and homocysteine levels were found, with respective contributions of 13.5%, 2.8%, 1.1%, 0.8%, 0.7%, and 0.7% [86].

There is much confusion with regard to lipoproteins and CIMT. Single-center studies indicate the relationship between higher CIMT and higher levels of total cholesterol (TC), LDL-C, lipoprotein (a), and non-HDL-cholesterol, as well as inverse associations with HDL-C; however, meta-analyses fail to show associations [62,85,86,87,88,89]. For example, in a study by Stamler et al. in a group of men aged 18 to 39 years, those with TC levels ≥ 6.21 mmol/L had a greater risk of CHD (2.15 to 3.63 times) and CV mortality (2.10 to 2.87 times) in comparison to individuals with TC < 5.17 mmol/L [90]. In this study, LDL-C, which is a classical atherogenic lipid, had a lower predictive value for the presence of carotid plaque than TC. The problem of lipids and atherosclerosis is much more complex, as there are many different fractions of lipoproteins that are atherogenic (i.e., very low-density lipoprotein cholesterol, intermediate-density lipoprotein cholesterol, or lipoprotein (a)) [90].

Of note, the accumulation of many various risk factors impacts overall CIMT and plaque parameters [91]. Many systemic inflammatory and thrombotic biomarkers are associated with increased CIMT and asymptomatic and symptomatic plaque incidence. Associations were proven for CIMT and interleukin-6 (IL-6), vascular cell adhesion molecule-1 (VCAM-1), Apolipoprotein E (ApoE), white blood cell (WBC) count, T lymphocytes, fibrin, and adiponectin [21,90]. Carotid plaque presence was associated with intercellular adhesion molecule 1 (ICAM-1), L-selectin, E-selectin, IL-1β, tumor necrosis factor α (TNF-α), lipoprotein phospholipase A2 (Lp-PLA2), WBC count, and mi-RNAs [90]. While transformation of asymptomatic into symptomatic carotid plaque was associated with levels of high-sensitivity C-reactive protein (hs-CRP), serum amyloid-A Protein (SAA), TNF-α, plasma-soluble urokinase plasminogen activator receptor (suPAR), matrix metalloproteinases (MPO-1, -2, -7, -9), tissue inhibitors of metalloproteinase (TIMP), ApoE, ApoA-I, Lp-PLA2, and miRNAs [92].

## 7. Additive Value of CIMT and Carotid Plaque to the Traditional Cardiovascular Risk Scoring Systems

As CIMT and carotid plaque are measures of atherosclerosis, it seems reasonable to combine established traditional risk scores with carotid imaging [93,94]. In a study by Elaid et al., 127 (37.8%) out of 336 initially ‘low -risk’ primary care patients (FRS event risk < 5% in 10 years) were re-classified as higher risk (>5%) when high CIMT (CIMT ≥ 75th percentile adjusted for age, gender, race, and presence of plaque) was found on ultrasound [95]. Plaque exceeding 1.5 mm was present in 17.3% of low-risk patients [95].

The risk calculators may integrate CIMT measurement with CV risk factors [86,93,94,95,96,97,98,99,100,101,102]. The ARIC study published an adjusted FRS calculator incorporating mean-maximum CIMT from six carotid segments and plaque assessment to determine the probability of MI or death from CHD within 10 years [96]. In that study including 13,145 individuals, approximately 23% were reclassified by adding CIMT and plaque information [96]. The addition of CIMT and plaque together to the traditional risk factors improved the prediction model from 0.742 for traditional risk factors to 0.755 for the CV risk factors, CIMT and plaque [96].

Recently, STRATEGY study assessed three scoring systems: the FRS, the Prospective Cardiovascular Münster Study Score (PROCAM), and the European Society of Cardiology SCORE in the context of possible additive CIMT value [85,92,95,96]. All scores correlated significantly with CIMT, but this correlation was only moderate [87]. The FRS correlated most strongly and predicted 27% of the CIMT variance in men and 20% in women [87].

The IMPROVE study, in a group of 3703 primary care patients aged 54–79 years with at least three CV risk factors, but free of any CV events prior to enrolment, evaluated the independence of carotid plaque thickness and the mean CIMT (measured in plaque-free areas bilaterally in the CCA, the CB, and the ICA) in CV risk stratification at 3 years follow-up [99]. In this study, both plaque and CIMT occurred as independent predictors of MACCE, with values of 1.98 (1.47 to 2.67) and 1.68 (1.23 to 2.29), respectively, as well as cerebrovascular events. However, only plaque was an independent predictor of coronary events like MI, sudden cardiac death (SCD), angina pectoris, and coronary revascularization [99]. The authors concluded that in reclassification analyses, CIMT and plaque significantly add to the FRS score [99]. In line, in the study by Gaibazzi et al., carotid plaques (not CIMT) and echocardiographic cardiac calcium were significant predictors of angiographic CAD in patients without prior CHD but with signs or symptoms suspect of CAD, again incrementally correlated to FRS [102].

Mitu et al. found a relationship between CIMT and arterial stiffness with SCORE, FRS, QRISK, and PROCAM in an asymptomatic population [100,101]. The SCORE risk correlated better with CIMT, while the FRS and QRISK seemed more specific for increased arterial stiffness parameters [100]. Of note, arterial stiffness proved its clinical value for MACCE in various clinical scenarios, e.g., in patients with aortic valve stenosis [103].

## 8. Primary and Secondary Care Population and MACCE

There is much evidence that higher CIMT corresponds to higher likelihood of MACCE. At least six primary care large-cohort prospective studies examined the predictive value of CIMT (without plaque) on MACCE (Table 4). In general, CIMT values in the highest range are associated with a 1.4- to 3.2-fold risk increase for MI, a 1.4- to 3.5-fold risk increase for IS, a 2.3- to 2.9-fold risk increase for CV death, and a 1.75-fold risk increase for SCD (Table 4) [11,12,15,16,17,18,19,104,105,106,107,108]. This was evidenced regardless of method used for CIMT calculation (mean-mean, mean-maximum, the CCA only, or combined CCA-CB-ICA) [12,13,15,104]. Importantly, in most studies, the ability of CIMT to predict future MACCE was independent of traditional risk factors [11,12,15,16,17,18,19,104,105,106,107,108]. 

There is much evidence that plaque presence is stronger predictor of MACCE than CIMT alone (Table 4) [11,59,94,108,109,110,111,112,113,114,115,116,117,118,119,120]. In a study comparing results of ARIC and CHS studies, the presence of plaque was associated with over 30% increased risk of SCD: 1.37 in the ARIC and 1.32 in the CHS [105]. In the Manhattan Study, carotid plaque thickness exceeding 1.9 mm was associated with a MACCE incidence risk of 2.8 (2.03 to 3.84), as compared to individuals with no plaque at all [110].

Further improvement in risk estimation may be gained by considering not only the largest identified plaque, but also the total plaque burden, plaque area, plaque score (a sum of all plaques heights), or a number of segments containing plaque in both carotid arteries (Table 4) [109,111,112,113,114,115,116,117,118,121]. According to some authors, the average of all the CIMTmax observed in each carotid segment (CIMTmean-max), is the variable that best describes the total plaque profile, and which has the best predictive power [21,118]. Additionally, carotid plaque burden measured by 3D ultrasound is highly correlated with CAC scores and predictive of MACCE (CV death, MI, and stroke) [111]. The High-Risk Plaque BioImage Study compared CIMT, carotid plaque burden, and maximum carotid plaque thickness in nearly 6000 individuals [111]. Both carotid plaque burden and carotid plaque thickness were predictive of primary and secondary MACCE, whereas CIMT was not [111].

The estimated added predictive value of carotid plaque thickness in comparison to traditional risk factors accounts respectively for 0.73 vs. 0.72 in the CHS study [116], 0.72 vs. 0.67 in Stork et al.’s study [113], and 0.90 vs. 0.88 in Prati et al.’s study [115], as well as 0.75 vs. 0.74 in the Tromso, ARIC, and Xie et al. studies [105,108,112]. The addition of the carotid plaque score to the established risk factors can significantly improve risk discrimination (C-index 0.746 vs. 0.726; *p* = 0.017) [114].

Importantly, plaque echogenicity, surface, angiogenesis, and size (volume and area) are all among risk factors for both cerebrovascular and cardiac events [121,123,124,125,126,127]. There is strong association between an increased risk of IS and plaques that are low echogenic (echolucent), ulcerated, with neovascularization, or containing mobile fragments with estimated respective risk of IS (HR, 95% CI): 3.99 (3.06 to 5.19), 3.58 (1.66 to 7.71), 9.68 (3.14 to 123.2), and 1.57 (1.02 to 2.41), respectively [125].

In contrast to primary prevention studies, there are only few studies that address role of CIMT/plaque assessment to calculate risk of MACCE recurrence. Although the issue is clinically relevant, the assessment of CV risk in patients with already known athero-oclussive disease at any arterial site (coronary, carotid, or other), or after index CV event (primary MI, IS, critical limb ischemia (CLI)) is not supported by the guidelines [55,125]. This attitude seems justified with regard to CIMT assessment (with exclusion of plaque parameters) and evidenced by studies of Yoon et al. and Tada et al. (Table 4) [23,122]. In contrast, in the study of Yoon et al. performed in 479 patients with index acute IS, carotid plaque (not mean CIMT-CCA) was associated with risk of secondary CV event (Table 4) [23].

In the study including 652 patients with angiographic stenosis ≥ 50% in at least one arterial territory (coronary, supra-aortic, renal, and/or lower extremity), who underwent a revascularization procedure for index lesion, a mean-max CIMT (plaque included) exceeding ≥ 1.25 mm (HR, 2.52; 95% CI, 1.5 to 4.24; *p* = 0.001) was associated with increased risk of MACCE, abdominal aortic aneurysm rupture, or development of new symptomatic lesions requiring revascularization [4]. In this study, inclusion of CIMT and plaque into the stratification model significantly improved the prediction of CV event risk [4]. Incremental value of mean-max CIMT plus plaque, TNF-α, and hs-CRP to traditional risk factors in risk stratification was also found in another study of patients with confirmed atherosclerosis [21]. Yet, the study by Tada et al. showed a combined risk for all-cause death, CV death, MI, IS, revascularization, heart failure of 3.38 (95% CI, 1.82 to 6.27) among 2035 patients diagnosed with atherosclerotic CVD [122].

## 9. Follow-Up of Changes in CIMT and Carotid Plaque Thickness with Multiple Assessments—Is It Worth It?

Serial assessment of CIMT change over time is considered a good method to monitor the natural progression of atherosclerosis in epidemiological studies and/or to assess the average response to treatment in clinical trials [27,40,41,44,116,121,122,128,129,130,131]. A major advantage of measuring carotid plaque burden is that progression/regression of plaque can be measured in clinically relevant time frames. The spatial resolution of carotid ultrasound is approximately 0.3 mm, and on average, CIMT changes by only 0.015 mm/year [128]. It is therefore not possible to reliably measure change in CIMT within an individual over short period of time. The consensus sample size for studies of effects of therapy on CIMT is 200 to 300 patients per group, followed for 2 years [27]. Thus, an appropriate time span is required between individual CIMT and plaque size assessments.

In the Malmo Diet and Cancer Study (MDCS) including 3426 primary care middle-aged Swedish patients, there was a cumulative relationship between traditional CV risk factors and CIMT progression rates during the 16-year follow-up period. The ORs of a high CIMT-CCA progression rate (>75th percentile) were 1.0 (reference), 1.4 (95% CI: 1.1 to 1.7), 1.7 (95% CI: 1.3 to 2.2), and 2.1 (95% CI: 1.4 to 3.1), respectively, for individuals with none, one, two, and three risk factors [44]. Favorable changes in systolic blood pressure, LDL-C, and HDL-C during over 15 years of follow-up decreased the CIMT progression rate in the CCA [44]. Interestingly, averaged CIMT progression rates were lower in the CCA (0.011 mm/year for men and 0.010 mm/year for women) but greater in the CB (0.036 mm/year for men and 0.030 mm/year for women) [44].

In a prospective study of a primary care population in the Cholesterol-Lowering Atherosclerosis Study trial, during an 8.8-year observation, Hodis et al. showed that the risk of coronary events was increased with the rate of CIMT progression (Table 5). The researchers observed ORs of coronary events of 1.0 (reference), 1.6, 2.3, and 2.8 for CIMT progression rates of less than 0.011, 0.011 to 0.017, 0.018 to 0.033, and greater than 0.033 mm/year, respectively [40]. In another study, CIMT progression predicted CV events in patients with type 2 diabetes [41].

A novel approach was recently proposed by the IMPROVE study to assess carotid CIMT progression [118]. In this study, the greatest value among the progressions of CIMTmax observed in the whole carotid tree identified focal increases of CIMT and was associated with cardiovascular risk (Table 5) [118].

In a secondary prevention population of 108 patients who had stent-supported angioplasty for symptomatic subclavian steal syndrome, followed for a mean of 4.8 years, Wrotniak et al. found CIMT progression of 0.060 mm/year to increase risk of MACCE and lesion progression by 22% (OR, 1.22; 95% CI, 1.02 to 1.46; *p* = 0.033) with a sensitivity of 75.0% and specificity of 61.8% [24]. In this study, despite medical treatment adhering to guidelines, atherosclerosis progression was found in 53 (49%), no change in 10 (9.3%), and regression in 45 (41.7%) patients [24].

Gacoń et al. demonstrated in a group of 215 patients admitted with acute coronary event, that patients with MACCE at follow-up, compared to MACCE-free subjects, had a greater annual CIMT progression rate either at first (0.024 ± 0.12 vs. 0.009 ± 0.16 mm/year; *p* < 0.001) or at subsequent follow-up ultrasound visits (0.050 ± 0.1 vs. 0.001 ± 0.1 mm/year; *p* < 0.001) [130]. Of note, initial CIMT values were similar in MACCE vs. MACCE-free patients (1.43 ± 0.40 vs. 1.45 ± 0.44 mm; *p* = 0.486) [130]. 

In Hirano et al.’s study including 240 patients with CHD confirmed on angiography, the average number of carotid plaques (≥1.1 mm of CIMTmax) at baseline was 2.5 ± 1.8 in a patient [128]. The change in plaque-IMTmax over 6 months ranged from −0.85 to 0.97 mm (mean, −0.006 ± 0.319 mm). The study showed that progression of carotid plaque-IMTmax over 6 months despite anti-atherosclerotic therapy was an independent predictor of future coronary events in CHD patients (Table 5) [133].

It is extremely important to understand that CIMT and plaque progression rate is non-linear [42,130]. Among that innumerous studies that were published, a study by Olmastroni et al. deserves particular attention as it is a large primary care cohort (1175 participants), with participants initially at low and intermediate CV risk with a prospective follow-up of 12 years, with the use of individual CIMT growth curve modeling [42]. Participants completed four visits with ultrasound examination, which proved that the rate of change in CIMT over time is a sign of the development of atherosclerosis, with periods of rapid and attenuated CIMT growth, which cannot be a priori assumed as linear [42]. In that study, the fastest mean and max CIMT growth was observed in patients between 50 and 70 years old. Of 966 subjects free from carotid atherosclerosis at baseline, 31.8% developed multifocal carotid atherosclerosis and 11.8% developed focal carotid atherosclerosis [42].

The non-linear response of atherosclerosis to so-called optimal medical treatment was also reported by study of Gacoń et al., including 466 secondary care patients [134]. In this study, regression of the mean-max CIMT (with inclusion of plaque thickness when present) was observed in 37.1% of the study group at the first ultrasound re-examination between month 12 and 24, and it went down to 26.6% at the second re-examination between month 24 and 36 [134]. The attenuated CIMT/plaque progression was independently associated with a reduced risk of MACCE (HR, 0.25; 95% CI, 0.15 to 0.42), MI (HR, 0.32; 95% CI, 0.20 to 0.51), IS (HR, 0.29; 95% CI, 0.18 to 0.45), and CV death (HR, 0.24; 95% CI, 0.15 to 0.40) [134]. In contrast, a carotid atherosclerosis progression of >0.056 mm/year was associated with increased risk of MACCE, however, with only moderate sensitivity and specificity of 53.2% and 72.2%, respectively (Table 5). Thus, achieving regression in CIMT and plaque thickness may constitute a better measure of treatment efficacy. 

## 10. CIMT and Carotid Plaque Changes in Response to Medical Treatment

CIMT was used in randomized clinical trials (RCT) to measure the effect of medical intervention, targeted at CV risk factor control, and the carotid atherosclerosis progression or regression, as possible modifiers of adverse CV outcomes [12,26,27,28,29,30,31,32,33,34,35,36]. Positive response to the medical intervention was defined as a measurable decrease in CIMT and carotid plaque values of the treated group compared to patients’ group, with treatment failure defined as CIMT or carotid plaque increase despite treatment in the context of future MACCE [37,38,39,40,41,42].

As effective interventions targeting pre-existing CVD, lifestyle and diet may reduce the risk of carotid atherosclerosis [85]. Huang et al. suggested that antihypertensive medication use may be the strongest modifiable predictor of slowing CIMT progression over time, especially when measurements are taken at the CB [135]. Overall, 8 out of 10 analyzed statin RCTs showed that conventional statins therapy are efficient and safe to decrease the rate of carotid atherosclerosis progression in the long term, and aggressive statins may provide superior efficacy for carotid atherosclerosis regression [34]. According to Wannanong et al., for assessment of response to anti-atherosclerotic therapy, measurement of total plaque volume is superior to both CIMT and total plaque area measurements [128]. This finding is in line with meta-analysis of seven studies including 361 patients receiving statin therapy, in which there was significant decrease in lipid-rich necrotic-core volume at >12 months (−9.9 mm^3^, 95% CI −8.9, −2.3); however, no significant reduction in carotid wall volume was seen on high-resolution carotid plaque MRI [136].

Conversely, according to the SAIP research group, the scientific bases for monitoring changes in single individuals are still not convincing [137]. First, Goldberger suggested caution in using CIMT as a surrogate endpoint of outcome in trials with statins, focused on CIMT progression/regression and MACCE incidence, although a smaller rate of change in CIMT was associated with a reduced MI incidence 0.82 (95% CI, 0.69 to 0.96; *p* = 0.018) [35]. Alas, there was no significant relationship between mean change in CIMT and nonfatal MI in RCTs [35]. Another large meta-analysis of 16 prospective studies performed by the PROG-IMT collaboration revealed a positive association between the mean CIMT-CCA and a 16% increase in CV risk, but no association between CIMT progression and CV events [132]. However, in this meta-analysis, the reproducibility between first and the second CIMT measurement was surprisingly low (correlation coefficient < 0.10), resulting in huge bias for data interpretation [138]. As consequence, the conclusion from meta-analyses of RCTs was that CIMT changes (regression or progression) did not correlate with changes in the incidence of MACCE induced by several drug treatments in different categories of subjects at intermediate to high CV risk [29]. 

This lack of associations between CIMT changes and clinical outcomes is surprising, as active medical treatment with either statins, calcium channel blockers, angiotensin-converting enzymes, or sartans was associated both with MACCE rate reduction as well as CIMT decrease in comparison to placebo groups [12,26,27,28,29,30,31,32,33,34,35,36,37,38].

To overcome existing confusion, a meta-analysis of 119 clinical trials involving 100,667 patients done by Willeit et al. shed some light on this puzzle [132]. Data from individual RCTs were systematized (Table 5). CIMT was assessed as the mean value at the CCA; if unavailable, the maximum value at the CCA or other CIMT measures were utilized. The primary outcome was a combined CVD endpoint defined as MI, stroke, revascularization procedures, or CV death. Authors estimated intervention effects on CIMT progression and incident CVD for each trial, before relating the two using a Bayesian meta-regression approach. This meticulous work resulted in conclusion that medical interventions reducing CIMT progression by only 0.01, 0.02, 0.03, or 0.04 mm/year would decrease the relative risks for CVD of 0.84 (95% CI, 0.75 to 0.93), 0.76 (95% CI, 0.67 to 0.85), 0.69 (95% CI, 0.59 to 0.79), or 0.63 (95% CI, 0.52 to 0.74), respectively [132].

## 11. Important Limitation for Comprehensive Data Analysis and Results Interpretation

Based on the experience in previous large-scale trials, there is a number of aspects that one may consider in designing a trial with CIMT as primary outcome parameter. For example, a major flaw in CIMT and carotid plaque measurements is the inter-observer and intra-observer reproducibility of measures. Although many recent studies demonstrated good agreements between intra-observer (between 91% and 97%) and inter-observer (between 88% and 91%) reliability of CIMT [120,122,139], data reproducibility must be assured.

An important limitation for comprehensive data analysis and results interpretation is the different methodology of CIMT and plaque measurements used in individual studies. That issue was clarified in the dedicated ASE and Mannheim recommendations [8,54,59].

Information on plaque changes in time carries useful information on treatment efficacy. Patients who are responders to medical treatment in terms of attenuation of carotid atherosclerosis growth have a decreased risk of MACCE. Therefore, last but not least, it is important to perform serial repeated carotid atherosclerotic burden measurements with appropriate period intervals between measurements, as atherosclerosis changes can fluctuate with periods of rapid and slow growth or regression. The single re-assessment of CIMT and plaque is a shortcoming. 

## 12. Perspectives for CIMT and Carotid Plaque on Ultrasound

The 2020 ASE recommendations for carotid plaque ultrasound suggested a stepwise approach to CV risk stratification adopted from Greenland et al. [140] and Piepoli et al. [124]. At baseline, carotid vascular ultrasound and subsequent 2D or 3D plaque quantification would be performed in the assessment of asymptomatic patients at low or intermediate risk according to an FRS and European SCORE. Patients with no plaque or carotid plaque thickness less than 1.5 mm would be considered as low risk. Patients with plaques thickness (CIMT) between 1.5 and 2.4 mm would be allocated to the intermediate risk class, while whose with plaques exceeding 2.4 mm would be considered in the high CV risk class with subsequent assessment of patient and plaque vulnerability (neovascularization and echolucency) [8].

In conclusion, CIMT and carotid plaque reflects atherosclerosis burden in the whole arterial tree. Incidence and severity of CV risk factors (both traditional and non-traditional) have an impact on CIMT thickness and plaque burden, and more importantly, they are responsible for the rate of carotid atherosclerosis progression. CIMT and carotid plaque may play an additive role in scoring systems evaluating CV risk. Thus, it appears reasonable to combine established risk scores with CIMT and plaque imaging. Both in the primary and the secondary care populations of patients, baseline parameters of CIMT and plaque thickness are associated with risk of future CV events. Aggressive medical treatment focused on CV risk factors’ elimination is associated with lesser progression of carotid atherosclerosis. However, whether medical interventions have an impact on the decreased risk of CV events through the reduction of CIMT and carotid plaque burden remains a matter of debate and needs further studies.

The authors of this review believe that averaged value of maximum CIMT with inclusion of maximum plaque thickness (when applicable) assessed at both the CCA, the CB, and the ICA is the best way to display atherosclerosis burden, and it well stratifies the CV risk and adverse events incidence both as baseline values and as a serial assessment. However, its clinical appliance should be matter of further investigations.

## Figures and Tables

**Table 1:** Normative values for CIMT. An overview of studies.

Study	Population	Number of Participants	Site of CIMT Assessment	CIMT Cut-Off	CIMT Determinant	CIMT Cut-Off
Lim, T.K., et al. 2008 [48]	Healthy individuals: no CVD, no hypertension, no diabetes, BMI < 30 kg/m^2^, and total cholesterol < 6 mmol/L.	137 women and men	Bilaterally, far wall of CCA and CB	Over 97.5th percentile of the distribution	CCA and age: 35–39 y.o. 40–49 y.o. 50–59 y.o. over 60 y.o.	0.60 mm 0.64 mm 0.71 mm 0.81 mm
					CB and age: 30–39 y.o. 40–49 y.o. 50–59 y.o. over 60 y.o.	0.83 mm 0.77 mm 0.85 mm 1.05 mm
Estíbaliz, J., et al. 2010 [49]	Healthy individuals with a BMI < 30 kg/m^2^, blood pressure < 160/90 mmHg, LDL-C < 160 mg/dL, HDL-C > 30 mg/dL, TG < 200 mg/dL, glycaemia < 125 mg/dL, creatinine < 2 mg/dL, or thyrotropin < 6 μU/mL.	74 women 64 men	Bilaterally, the average CIMT of the CCA, CB, and ICA	Over 75th percentile of the distribution	Female and age: <25 y.o. 25–34 y.o. 35–44 y.o. 45–54 y.o. 55–64 y.o. >64 y.o. Male and age: <25 y.o. 25–34 y.o. 35–44 y.o. 45–54 y.o. 55–64 y.o. >64 y.o.	0.52 mm 0.58 mm 0.65 mm 0.70 mm 0.80 mm 0.93 mm 0.59 mm 0.67 mm 0.66 mm 0.72 mm 0.81 mm 0.95 mm
Diaz, A., et al. 2018 [50]	Healthy individuals: BP < 140/90 mmHg in adults and < 90th percentile in younger subjects; no history of CVD, pulmonary, or renal disease; not taking antihyperlipidemic, blood lowering, or antidiabetic drugs; glycaemia < 110 mg/dL; total cholesterol <200 mg/dL; TG < 150 mg/dL and < 130 mg/dL for adults and subjects between 10 to 17 years, respectively.	391 women 621 men	Bilaterally, averaged far wall of the CCA	Over 75th percentile of the distribution	Female and age: <20 y.o. 25; 35 y.o. 40; 50 y.o. 55; 65 y.o. ≥70 y.o. Male and age: <20 y.o. 25; 35 y.o. 40; 50 y.o. 55; 65 y.o. ≥70 y.o.	0.47 mm 0.49; 0.55 mm 0.58; 0.67 mm 0.72; 0.83 mm 0.89 mm 0.49 mm 0.51; 0.56 mm 0.60; 0.68 mm 0.72; 0.83 mm 0.89 mm
Engelen, L., et al. 2013 [51]	24 research centers worldwide. Individuals without CVD or CV risk factors (BP < 140/90 mmHg), no current smoking, glucose < 7.0 and/or post-load plasma glucose < 11.0 mmol/L, total cholesterol < 6.2 mmol/L, HDL-C > 1.17 mmol/L (for men) and > 1.30 mmol/L (for women), BMI < 30 kg/m^2^, and no BP-, lipid-, and/or glucose-lowering medication	2241 women 1993 men	Bilaterally, averaged far wall of the CCA	Over 75th percentile of the distribution	Female and age: <20 y.o. 25; 35 y.o. 40; 50 y.o. 55; 65 y.o. 70; 80 y.o. ≥85 y.o. Male and age: <20 y.o. 25; 35 y.o. 40; 50 y.o. 55; 65 y.o. 70; 80 y.o. ≥85 y.o.	0.47 mm 0.50; 0.55 mm 0.58; 0.64 mm 0.66; 0.72 mm 0.75; 0.80 mm 0.83 mm 0.48 mm 0.51; 0.57 mm 0.59; 0.65 mm 0.68; 0.74 mm 0.80; 0.83 mm 0.86 mm
Randrianarisoa, E., et al. 2015 [52]	Healthy individuals: no CVD, no classic CV risk factors, and exclusion of metabolic syndrome, subclinical inflammation, insulin resistance, abnormal the body fat distribution, and prediabetes	428 women 373 men	Bilaterally, far wall of the CCA	Over 90% limits of the distribution	Female and age: 18–29 y.o. 30–39 y.o. 40–49 y.o. 50–59 y.o. Male and age: 18–29 y.o. 30–39 y.o. 40–49 y.o. 50–59 y.o.	0.47 mm 0.59 mm 0.67 mm 0.70 mm 0.47 mm 0.62 mm 0.72 mm 0.80 mm

BMI, body mass index; BP, blood pressure; CB, carotid bulb; CCA, common carotid artery; CIMT, carotid intima-media thickness; CV, cardiovascular; CVD, cardiovascular disease; HDL-C, high-density lipoprotein cholesterol; ICA, internal carotid artery; LDL-C, low-density lipoprotein cholesterol; TG, triglycerides.

**Table 2:** An overview on CIMT and carotid plaque definitions and acquisition techniques.

Study	CIMT Assessment	Carotid Plaque
	Definition, Acquisition Technique	Acquisition Technique, Definition
Mannheim IMT Consensus, 2012 [59]	Definition: CIMT is a double-line pattern visualized by ultrasound on both walls of the CCA in a longitudinal image, which consist of the leading edges of two anatomical boundaries: the lumen-intima and media-adventitia interfaces (Figure 1). Acquisition: High-resolution B-mode system with linear transducers at frequencies > 7 MHz, log gain compensation of app. 60 dB. Gain settings adjusted to obtain a symmetrical brightness on the near and far wall to eliminate artifacts in a longitudinal view lateral position. A long 10 mm length of a straight arterial segment is required for reproducible serial measurements. CIMT measurement within a region free of plaque with a clearly identified double-line pattern, preferably on the far wall of the CCA at least 5 mm below its end. CIMT can be measured at the CB or ICA in a region free of plaque, on a shorter length, taking caution of the large interindividual variability. These values must be recorded separately. CIMT measurements options include the mean, maximum, composite measures from both sides, and different arterial sites. Mean CIMT values averaged across the entire distance are less susceptible to outliers. The maximal CIMT may reflect more advanced stages with focal thickening or plaque formation.	Definition: Plaques are focal structures encroaching into the arterial lumen of at least 0.5 mm or 50% of the surrounding CIMT, or demonstrating a thickness of >1.5 mm as measured from the intima-lumen interface to the media-adventitia interface (Figure 1). Acquisition technique: Plaque location, thickness, area, and number scanned in longitudinal and cross-sections must be recorded. For plaque, a maximal thickness requires demonstration from 2 different angles of insonation, in longitudinal and cross-sectional views. The incremental value of recording texture (density, echogenicity, shadow) remains uncertain pending more research.
American Society of Echocardiography, 2008 [54] and 2020 [8]	Definition: not given Acquisition technique: B-mode imaging preferred over M-mode. Ultrasound system with a linear-array transducer at frequencies > 7 MHz. CIMT imaging (3–5 beat cine-loop and optimized R-wave gated still frames at each angle).Distal 1 cm of each CCA. Use of a semiautomated border detection program with validated accuracy. Scanning protocols from observational studies with published nomograms may be used if they are more germane to the age, sex, and race/ethnicity of the clinical population being investigated; however, the clinical laboratory must have sufficient expertise to perform them accurately and reproducibly. Use of values from clinically referred populations are discouraged, because of the high likelihood of referral bias and inaccurate risk estimates. Limiting CIMT measurements to the far wall of the CCA is the preferred strategy. Interpretation of carotid ultrasound studies for CVD risk assessment: Mean CIMT values from the far wall of the right and left CCAs (mean-mean) should be reported. Use of additional segments or maximum values is an alternative if there is local expertise and normative values with published associations to CVD risk are reported. Mean-mean values are more reproducible because multiple points along the traced segment are averaged, but are less sensitive to change. Mean-maximum values are more sensitive to change, but less reproducible. Evaluating for the presence or absence of plaque in conjunction with measuring CIMT-CCA offers a better representation of subclinical vascular disease and CVD risk than only measuring CIMT.	Definition: Carotid arterial plaque visualized with or without use of an ultrasound enhancing agent is defined as: (1) any focal thickening thought to be atherosclerotic in origin and encroaching into the lumen of any segment of the carotid artery (protuberant-type plaque), or (2) in the case of diffuse vessel wall atherosclerosis, when CIMT measures ≥ 1.5 mm in any segment of the carotid artery (diffuse-type plaque). Carotid plaque grading: Grade 0: no carotid plaque; Grade I: focal protuberant thickening of vessel wall < 1.5 mm; Grade II: focal protuberant plaque between 1.5 and 2.4 mm height, or diffuse thickening of the vessel wall between 1.5 and 2.4 mm; Grade III: either protuberant or diffuse thickening above 2.4 mm. Repeat measurements are not recommended unless the Grade and CIMT meets criteria for diffuse-type plaque (Grades II or III, and CIMT ≥ 1.5 mm) in which case it is a plaque equivalent.
		Acquisition technique: 2D techniques for quantifying plaque as initial approach with giving maximum plaque thickness. It should be measured from the side in which a plaque lesion is detected (unilateral) or from both the right and left carotid arterial segments (bilateral) using a caliper placed at the adventitial plane and extending into the center of the lumen at right angles to the vessel wall. For standardization, this measurement should be taken from segments of the long and short axis. 2D plaque area: the measurement should begin from medial-adventitial plane for the purposes of standardization. The quantification of plaque volume for an individual plaque lesion is recommended when required (e.g., morphologic assessment, serial assessment, or pre-operative consideration), using either the stacked-contour method or specialized semi-automated tools. 3D plaque volume: the quantification of right and/or left carotid plaque volume using 3D ultrasound for cardiovascular risk stratification with a single-plaque or single-region report, or a full-vessel protocol report.

2D, 2-dimentional; 3D, 3-dimensional.

**Table 3:** Meta-analyses on the increased CIMT and carotid plaque incidence with cardiovascular risk factors.

	Song et al. 2020 [62]	Ji et al. 2019 [85]
Risk Factor	Pooled Data OR (95% CI)	Pooled Data OR (95% CI)
Increased CIMT (>1.0 mm) Age Female sex Current smoking Diabetes Hypertension Dyslipidemia	2.71 (2.07–3.55) 0.49 (0.38–0.64) 1.76 (1.34–2.30) 2.23 (1.48–3.36)	N/A N/A N/A N/A
	1.55 (1.03–2.34)	2.60 (1.33–5.08)
	0.90 (0.65–1.25)	N/A
Carotid plaque Age Female sex Current smoking Diabetes Hypertension Dyslipidemia HDL-C LDL-C Hypertriglyceridemia Hyperuricemia Hyperhomocysteinemia Metabolic syndrome	1.79 (0.93–3.43) 0.55 (0.33–0.94)	- -
	1.70 (1.41–2.04) 1.45 (1.12–1.90) 1.75 (1.44–2.13) - 0.46 (0.21–0.99) - - - - -	1.41 (1.08–1.87) 1.31 (1.13–1.53) 1.81 (1.55–2.13) 1.20 (0.80–1.82) 1.28 (0.99–1.67) 1.11 (1.08–1.13) 1.33 (1.14–1.55) 1.57 (1.11–2.22) 1.88 (1.19–2.95) 1.71 (1.10–2.66)

**Table 4:** Overview of studies on the relationship between CIMT, carotid plaque, and MACCE in primary and secondary cardiovascular risk prevention individuals.

Study	CIMT Measure	Interpretation	Participants Number, Type	Follow-Up (Years)	Outcome	HR (95% CI)
**Primary Cardiovascular Risk Prevention**						
CIMT studies CAPS [12]	Mean-CCA	4th vs. 1st quartile 4th vs. 1st quartile 4th vs. 1st quartile	5052, PC 5052, PC 5052, PC	4.2 4.2 4.2	MI IS CV death	2.3 (0.9–6.3) 2.3 (1.4–3.8) 3.2 (2.0–5.1)
CHS [13]	Max-CCA	5th vs. 1st quartile 5th vs. 1st quartile 4th vs. 1st quartile	4476, PC 4476, PC 5555, PC	6.2 6.2 13.1	MI IS SCD	3.2 (2.0–5.1) 2.8 (1.8–4.2) 1.75(1.25–2.51)
MDCS [15]	Mean-CCA	3rd vs. 1st tertile 3rd vs. 1st tertile	5163, PC 5163, PC	7.0 7.0	MI IS	2.1 (1.2–3.4) 3.0 (1.6–5.7)
KIDH [16]	Max-CCA	≥1 mm vs. < 1.0 mm	1275, PC	1.0	MI	2.2 (0.7–6.7)
Rotterdam [17]	Mean-CCA	Per 0.16 mm, 1SD Per 0.16 mm, 1SD	1566, PC 1566, PC	2.7 2.7	MI IS	1.4 (1.2–1.8) 1.4 (1.3–1.8)
LILAC [104]	Mean-CCA, CB, ICA	Per 0.3 mm	298, PC	3.1	CV death	2.9 (1.0–6.8)
Carotid plaque studies CHS [105]	Plaque presence	Plaque vs. no plaque	5555, PC	13.1	SCD	1.32 (1.04–1.67)
ARIC [18,19]	Mean-CCA- CB-ICA+plaque	>1 mm > 1 mm > 1 mm > 1 mm	10841, PC 10841, PC 14214, PC 14214, PC	5.2 5.2 7.2 7.2	MI MI IS IS	M: 1.8 (1.3–2.7) F: 5.1 (3.1–8.4) M: 2.0 (1.2–3.1) F: 3.3 (1.9–5.8)
ARIC [105]	Plaque (>1.5 mm) Mean-CCA-CB-ICA+plaque	Plaque vs. no plaque 4th vs. 1st quartile	15307, PC 15307, PC	23.5 23.5	SCD SCD	1.37 (1.13–1.67) 1.64 (1.15–2.63)
Ali J.S. et al. [107]	Mean-CCA- CB-ICA+plaque	4th vs. 1st quartile	706, PC	4.78	MI, IS, TIA, revascularization	5.8 (1.3–26.60)
TROMSO [107,108]	Plaque area	4th quartile of plaque area vs. no plaque	3240 M, PC 3344 F, PC 2989 M, PC 3237 F, PC	10 10 5.4 5.4	IS IS MI MI	1.73 (1.19–2.52) 1.62 (1.04–2.53) 1.56 (1.04–2.36) 3.95 (2.16–7.19)
Manhattan Study [110]	Max. plaque thickness	Plaque > 1.9 mm vs. no plaque	2189, PC	6.9	MI, IS, CV death	2.80 (2.04–3.84)
Biolmage Study [111]	3D Plaque max thickness	3rd tertile vs. no plaque	5808, PC	2.7	MI, IS, CV death	2.36 (1.13–4.92)
Xie et al. [112]	Sum of segments with plaque	≥ 3 segments with plaque(s)	3258, PC	5	CHD, IS	2.43 (1.20–4.93)
Stork et al. [113]	Sum of all plaques areas	By number of plaques: < 2; 2 to 4; > 4	403, PC	4	CV death	1.85 (1.14–3.01)
ARCO study [109]	Total plaque area	3rd tertile of plaque area vs. no plaque	2842, PC	5.9	MI, IS, CV death	21.4 (2.8–163)
MESA study [114]	Total plaque score	Plaque score per 1 SD	6814, PC	11.3	CV disease CHD IS	1.27 (1.16–1.40) 1.35 (1.21–1.51) 1.15 (0.98–1.35)
Yang, C.W.; et al. [120]	Mean-max CIMT-CCA, CB, ICA plus plaque	CIMT/plaque >2 mm vs. <1 mm CIMT >1 mm vs. <1 mm	2956, PC 2956, PC	9.41 9.41	All-cause death All-cause death	1.79 (1.07–3.00) 1.65 (1.21–2.32)
**Secondary cardiovascular risk prevention**						
CIMT studies						
Yoon et al. [23]	mean CCA	mean	479, AIS	9	MI, IS, CLI, CV death	2.21 (0.80–6.09)
Tada et al. [122]	mean-max CCA	mean	2035, ASCVD	4	All-cause death CV death, MI, stroke, HF, revascularization	0.89 (0.52–1.49)
Carotid plaque studies						
Kolkenbeck-Ruh et al. [22]	Plaque thickness	CIMT plus plaque	473, CLI or IS479, controls	n/a	IS vs. controls CLI vs. controls	<60 years, HR 20.8 to 28.4 (7.24–111) >50 years, HR 5.61 to 8.85 (1.77–25.4)
Kabłak-Ziembicka et al. [4]	Mean-max CIMT-CCA, CB, ICA plus maximum plaque thickness	Mean CIMT/plaque ≥ 1.25 mm (3,4 vs. 1,2 quartile)	652, Confirmed CHD 125, PC	5	MI, IS, CLI, CV death	2.52 (1.50–4.24)
Yoon et al. [23]	Plaque presence (Mannheim definition)	Any vs. no plaque	479, AIS	9	MI, IS, CLI, CV death	1.70 (1.14–2.53)
Tada et al. [122]	Carotid plaque score	top quintile vs. bottom quintile	2035, ASCVD	4	All-cause death, CV death, MI, IS, HF, revascularization	3.38 (1.82–6.27)

AIS, acute ischemic stroke; ASCVD, atherosclerotic cardiovascular disease; CLI, critical limb ischemia; CV, cardiovascular; F, female gender; HF, heart failure; IS, ischemic stroke; M, male gender; MI, myocardial infarction; PC, primary care subjects; SCD, sudden cardiac death; SSSS, symptomatic subclavian steal syndrome.

**Table 5:** The relationship between CIMT or carotid plaque progression over time and MACCE.

Study	CIMT Measure	Interpretation Progression Rate, mm/Year	Participants Number, Type	Follow-Up (Years)	Outcome	HR (95% CI)
**Primary cardiovascular risk prevention**						
CIMT studies Hodis, et al. [40]	Mean-CCA	< 0.011, 0.011 to 0.017, 0.018 to 0.033, > 0.033	188, PC	8.8	Coronary events	Ref. 1.0 1.6 (0.9–6.3) 2.3 (1.4–3.8) 2.8 (1.8–4.2)
IMPROVE study [118]	CIMT-CCA, BC, ICA	Fastest CIMT change > 0.026 mm/year	3482, CRF ≥ 3	1.3	MI, SCD, CV death, stroke, TIA	1.98 (1.47–2.67)
Okayama, et al. [41]	CIMT-CCA at baseline and at least 2 more times	Median CIMT change > 0.011 mm/year	342, diabetes	7.6	CV death, MI, IS	2.24 (1.25–4.03)
Willeit et al. [132] Meta-analysis of 119 clinical trials with medical agents	CIMT-CCA, mean or max	CIMT regression of: 0.01 mm/year 0.02 mm/year 0.03 mm/year 0.04 mm/year	100667, CRF	n/a	MI, stroke, CV death, revascularization	0.84 (0.75–0.93) 0.76 (0.67–0.85) 0.69 (0.59–0.79) 0.63 (0.52–0.74)
Carotid plaque studies Wannarong, T., et al. [128]	CIMT, TPA, TPV at baseline and after 1 year	By tertiles of change	349, PC	3.17	Death, stroke, TIA, MI	CIMT: *P* = 0.455 TPA: *P* = 0.143 TPV: *P* < 0.001
**Secondary cardiovascular risk prevention**						
Carotid plaque studies						
Wrotniak et al. [24]	Mean-max CIMT-CCA, CB, ICA plus plaque thickness, 2nd exam at M36-42	> 0.060 mm/year	108, SSSS	4.8	CV death, MI, IS, revascularization	1.22 (1.02–1.46)

Gacoń, J et al. [130]	Mean-max CIMT-CCA, CB, ICA plus plaque thickness, exams at yearly intervals	CIMT progression rate > 0.003 mm/year	215, ACS	4.4	MI, IS, CV death, new onset angina revascularization	3.0 (1.5–6.02)
Hirano et al. [133]	Max carotid plaqu eat baseline, 2nd exam after 6M	Per 0.1 mm increase over 6 months	240, CHD	3	Cardiac death, MI, UA with revascularization	1.21 (1.10–1.33)
Gacoń, J. et al. [134]	Mean-max CIMT CCA, CB, ICA plus plaque thickness, 2nd exam between M12-24, 3rd exam between M24-36	Mean CIMT/plaque ≥ 0.056 mm/year Any CIMT/plaque regression	466, ASCVD 466, ASCVD	3.5 3.5	MI, IS, CV death MI, IS, CV death	1.22 (1.03–1.44) 0.25 (0.14–0.32)

ACS, acute coronary syndrome; ASCVD, atherosclerotic cardiovascular disease, defined as lesions in at least one major arterial territory including coronary, carotid, renal, or lower extremity arteries exceeding 50% lumen reduction; CRF, cardiovascular risk factors; IS, ischemic stroke; M, month; MI, myocardial infarction; TPA, total plaque area; TPV, total plaque volume; UA, unstable angina.

## Data Availability

The data presented in this study are available on request from the corresponding author. The data are not publicly available due to privacy.
